# Differential development of oil granulomas induced by pristane injection in galectin-3 deficient mice

**DOI:** 10.1186/s12865-015-0133-9

**Published:** 2015-11-14

**Authors:** Camila Brand, Thayse Pinheiro da Costa, Emerson Soares Bernardes, Camila Maria Longo Machado, Leonardo Rodrigues Andrade, Roger Chammas, Felipe Leite de Oliveira, Márcia Cury El-Cheikh

**Affiliations:** Laboratório de Proliferação e Diferenciação Celular, Programa de Ciências Morfológicas, Instituto de Ciências Biomédicas, Universidade Federal do Rio de Janeiro, Rio de Janeiro, Brasil; Centro de Radiofarmácia, Instituto de Pesquisas Energéticas Nucleares (IPEN), São Paulo, Brazil; Laboratório de Oncologia Experimental-LIM24, Centro de Investigação Translacional em Oncologia, Instituto do Câncer do Estado de São Paulo, Departamento de Radiologia e Oncologia, Faculdade de Medicina, Universidade de São Paulo, São Paulo, Brasil; Laboratório de Investigação Médica Radioisótopos-LIM43, Departamento de Radiologia e Oncologia, Faculdade de Medicina, Universidade de São Paulo, São Paulo, Brasil; Laboratório de Biomineralização, Instituto de Ciências Biomédicas, Universidade Federal do Rio de Janeiro, Rio de Janeiro, Brasil; Faculdade de Medicina da Universidade de São Paulo, Instituto do Câncer do Estado de São Paulo, São Paulo, SP Brasil

**Keywords:** Pristane, Oil granuloma, Bone marrow, Peritoneal cavity, Inflammation

## Abstract

**Background:**

Galectin-3 is known to be a lectin that plays an important role in inflammatory processes, acting as pro-inflammatory mediator in activation and migration of neutrophils and macrophages, as well as in the phagocytic function of these cells. The injection of mineral oils into the peritoneal cavity of mice, such as 2, 6, 10, 14-tetramethylpentadecane (pristane), induce a chronic granulomatous inflammatory reaction which is rich in macrophages, B cells and peritoneal plasma cells known as oil granuloma. In addition, this inflammatory microenvironment provided by oil granulomas is also an important site of plasmacytoma induction, which are dependent on cytokine production and cellular mobilization.

Here, we have analyzed the role of galectin-3 in inflammatory cells mobilization and organization after pristane injection characterizing granulomatous reaction through the formation of oil granulomas.

**Results:**

In galectin-3 deficient mice (gal-3^−/−^), the mobilization of inflammatory cells, between peritoneal cavity and bone marrow, was responsible for the formation of disorganized oil granulomas, which presented scattered cells, large necrotic areas and low amounts of extracellular matrix. The production of inflammatory cytokines partially explained the distribution of cells through peritoneal cavity, since high levels of IL-6 in gal-3^−/−^ mice led to drastically reduction of B1 cells. The previous pro-inflammatory status of these animals also explains the excess of cell death and disruption of oil granulomas architecture.

**Conclusions:**

Our data indicate, for the first time, that the disruption in the inflammatory cells migration in the absence of galectin-3 is a crucial event in the formation and organization of oil granulomas.

**Electronic supplementary material:**

The online version of this article (doi:10.1186/s12865-015-0133-9) contains supplementary material, which is available to authorized users.

## Background

Chronic inflammation represents a heterogeneous group of physiological conditions that may affect virtually any organ or system. Histologically, the chronic inflammation exhibits a predominance of mononuclear cells, such as macrophages, lymphocytes and plasma cells. In contrast, neutrophils are relevant for the initial phases of the inflammatory process [1, 2]. The stromal *milieu* (cellular and matrix components) regulates leukocyte production and recruitment from the bone marrow to the site of the inflammatory stimulus, determining the outcome of the process. However, the production of bone marrow cells, including neutrophils, is also largely regulated by the rate of apoptosis of hematopoietic cells in the site of inflammation in a T cell dependent-manner [3, 4].

In this context, continuous and unbalanced recruitment of inflammatory cells such as neutrophils, monocytes, lymphocytes and dendritic cells, from the bone marrow or adjacent tissues, toward the chronic site of stimulus can be determinant to the development of several immune responses [5].

Chronic inflammation can be triggered by long-lasting exposure to naturally occurring hydrocarbon oils such as pristane (2, 6, 10, 14-tetramethylpentadecane, TMPD) [6], which is not metabolized and causes severe physiological consequences to mammals [7]. Although, the clinical significance of these lesions in the development of an infection or neoplasia remains unknown. Epidemiologic studies suggest that occupational exposure to hydrocarbon oils or petroleum waste is associated to rheumatoid arthritis and systemic lupus erythematous [8, 9]. In experimental mouse models, pristane induces a continuous intraperitoneal chronic inflammation followed by oil-granuloma formation and plasma cell tumors, known as plasmacytoma [10–12]. These granulomas are frequently adhered to peritoneal structures correlated to vigorous immune response, autoantibody production and clinical manifestations resembling systemic lupus erythematous [13].

Pristane-induced granulomas are characterized by individual and clustered cells adhered to the mesentery [14] and other peritoneal tissues often covered by mesothelial cells. Angiogenesis in these structures is also established by terminal mesenteric vessels. Moreover, the structure of oil granulomas is regulated by several cytokines, such as IL-6, IFNs, IL-2 and TNF-α [7, 15–17]. These oil granulomas are considered tertiary lymphoid tissues and are enriched by terminally differentiated plasma cells, macrophages and resident self-renewing peritoneal B-1 lymphocytes. In addition, they may be putative targets to understand plasmacytoma development [11, 18]. Although oil granulomas have been well characterized, the role of the first recruited cells to the peritoneal microenvironment and their participation in the organization of the granuloma are not well understood.

Galectin-3, a β-galactoside binding protein, modulates both chronic inflammation and fibrogranulomatous reaction [19], regulating cell-cell and cell-extracellular matrix interactions in healthy and pathological conditions [20–23]. Galectin-3 deficient mice (Gal-3^−/−^ mice) have a delayed monocyte differentiation into macrophages and neutrophil mobilization [24]. On the other hand, these mice have a significant plasmacytogenesis in secondary lymphoid tissues. In addition, the mesenteric membranes histologically correlated with disturbances in B lymphocyte and plasma cell niches [25–27].

In this study, we investigated the histological organization of pristane-induced oil granulomas in the absence of galectin-3. The kinect of cell mobilization and oil granuloma formation were analyzed in different time points of the inflammatory process. In the absence of galectin-3, the organization of oil granulomas was dependent on both bone marrow and peritoneal cavity cells, leading to a necrotic tissue, extracellular matrix disruption and disorganized cellular distribution. On the other hand, oil granulomas formed in the presence of galectin-3, did not present intense cell death, favoring maintenance and amplification of hematopoietic cells, mainly granulocytes.

## Results

### Cellularity in galectin-3^−/−^ mice is influenced by pristane injection and affects lymphocyte subpopulations

**Fig. 1:**
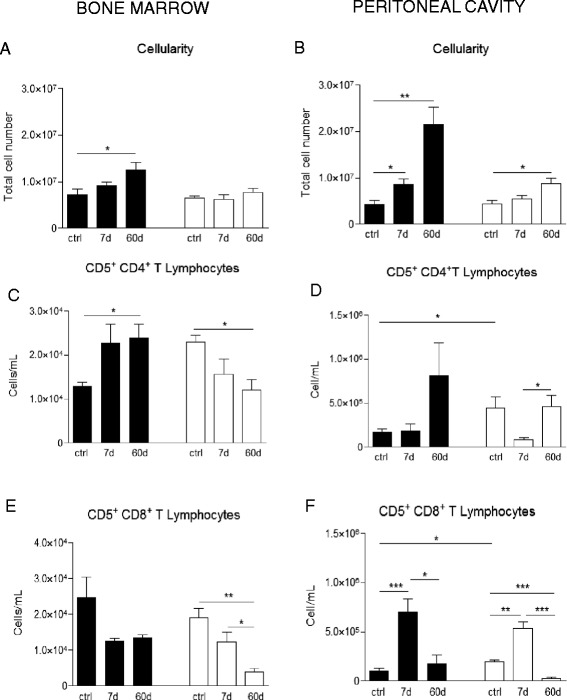
Quantification of T lymphocyte subpopulations in the bone marrow and peritoneal cavity after injection of pristane. Sixty days after injection of pristane, total cell number of bone marrow in WT animals were increased, differently of cellularity in gal-3^−/−^ mice, which remained constant compared to control group of animals. **a** In peritoneal cavity of both animals cellularity was increased, but in a lower level in gal-3^−/−^ mice compared to WT animals. **b** T lymphocyte cell subsets in the bone marrow and peritoneal cavity were quantified by flow cytometry and we analyzed CD4^+^ and CD8^+^ T lymphocytes in the bone marrow (**c** and **d**, respectivelly) and in peritoneal cavity (**e** and **f**, respectivelly). Data are reported as means ± standard error and are representative of five animals of each group. (*, *P* < 0.05). Black bars: WT animals; white bars: gal-3^−/−^ animals

Considering that galectin-3 deficient mice present a delay in hematopoietic cells differentiation and mobilization, we investigated the bone marrow and peritoneal cavity at 7 days (the beginning of cell mobilization) and 60 days (structurally organized inflammatory tissues attached to mesentery) after pristane injection, comparing to non-injected control animals. As we can observe in Fig. 1a, bone marrow cellularity in WT mice was gradually increased during the treatment, reflecting increased cellularity in the peritoneal cavity. These results suggest the continuous mobilization of inflammatory cells between bone marrow and peritoneal cavity. In gal-3^−/−^ mice, on the other hand, the bone marrow cellularity did not change, despite greater cellularity in peritoneal cavity, when compared to control group (Fig. 1a and b). Even with the increase of the total number of cells in the peritoneal cavity in gal-3^−/−^ animals, the cellularity was still reduced when compared to WT mice (Fig. 1a and b).

Lymphoid compartment along pristane injection presented differences in CD4^+^ and CD8^+^ T lymphocyte populations, both in bone marrow and peritoneal cavity. In the absence of gal-3, CD5 + CD4^+^ T cells were drastically reduced in bone marrow at 60 days after pristane injection (Fig. 1c and d). However, this cell population was not observed in peritoneal cavity, where CD5 + CD4^+^ T lymphocytes presented an initial decrease at day 7, but recovered at day 60 to initial numbers compared to gal-3^−/−^ control mice (Fig. 1d). On the other hand, CD5 + CD8+ T lymphocytes presented an opposite behavior, with initial increase, and then, returning to initial total values after 60 days when compared to control group (Fig. 1e). In contrast to gal-3^−/−^, WT animals presented an increase in CD5 + CD4^+^ T lymphocytes in bone marrow (Fig. 1c), accompanied by the increase of this population in the peritoneal cavity (Fig. 1d). However, no significant difference was observed in bone marrow CD5 + CD8^+^ T cells (Fig. 1e). In peritoneal cavity, CD5 + CD8^+^ T cells behavior was similar to that observed in gal-3^−/−^ mice (Fig. 1f).

**Fig. 2:**
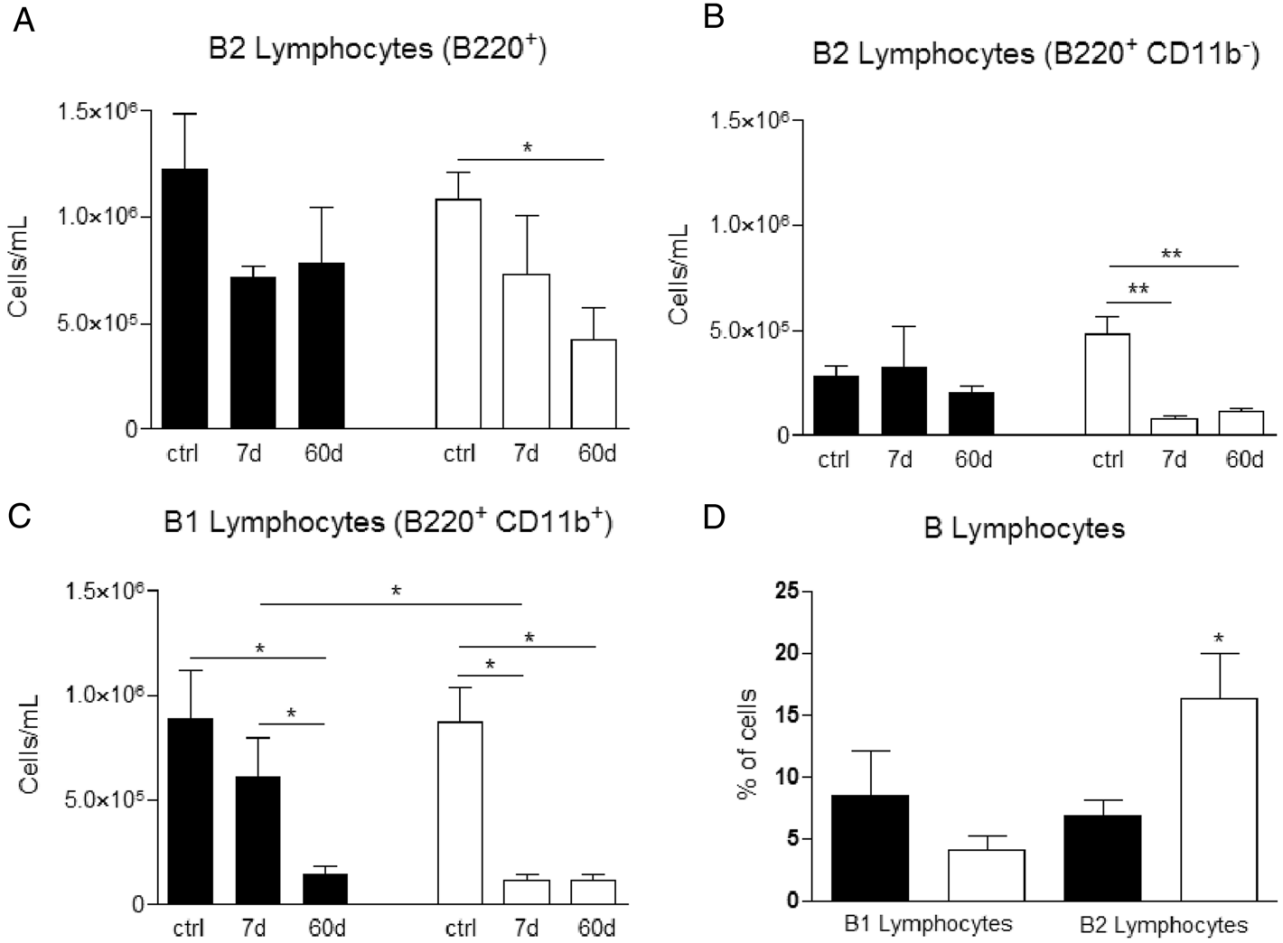
Quantification of B lymphocyte subpopulations in the bone marrow, peritoneal cavity and oil granuloma after injection of pristane. B lymphocyte cell subsets in the bone marrow, peritoneal cavity and dissociated oil granulomas were quantified by flow cytometry and we analyzed B200^+^ B cells in bone marrow (**a**), B2 (B220^+^ CD11b^−^ cells) and B1 (B220^+^ CD11b^+^ cells) lymphocytes in peritoneal cavity (**b** and **c**, respectivelly). B1 and B2 lymphocytes were also analyzed in dissociated oil granulomas. **d** Data are reported as means ± standard error and are representative of five animals of each group. (*, *P* < 0.05). Black bars: WT animals; white bars: gal-3^−/−^ animals

Total bone marrow B220+ B lymphocytes in gal-3^−/−^ animals were quite decreased at 60 days after pristane injection. In contrast, WT mice did not present any reduction presenting no significant differences when compared to control group (Fig. 2a). In the peritoneal cavity of WT animals no significant differences in B2 lymphocytes were observed, but in the absence of gal-3, these cells presented a drastic decrease at 7 days post injection. These differences were still maintained upon 60 days (Fig. 2b). The same pattern was observed for B1 cells in the peritoneal cavity in the absence of gal-3 already 7 days post pristane injection (Fig. 2c). Although in galectin-3 deficient mice B2 and B1 lymphocytes were found systemic and locally modified, when cell analysis of oil granulomas were performed after enzymatic dissociation we observed an increase in the total number only in the B2 lymphocytes (Fig. 2d).

### The pattern of granulocyte response is modified in the absence of galectin-3 and influences the oil granuloma composition

**Fig. 3:**
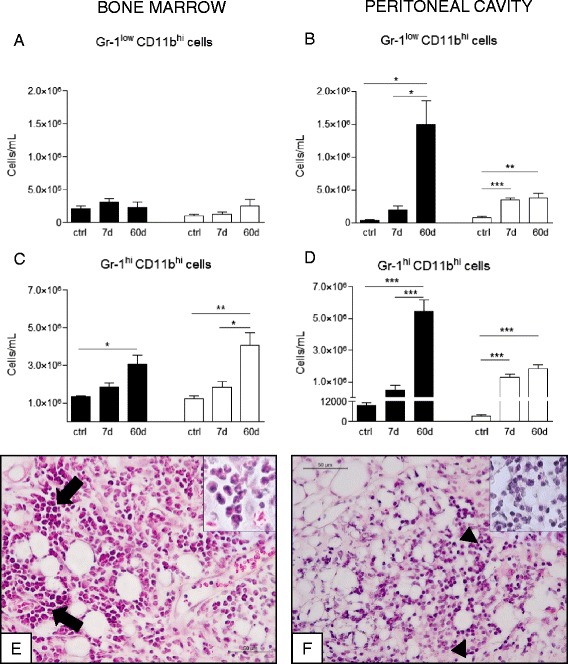
Quantification of granulocyte subpopulations in the bone marrow, peritoneal cavity and oil granuloma after injection of pristane. Granulocyte cell subsets in the bone marrow, peritoneal cavity and oil granulomas were quantified by flow cytometry and characterized by morphological analysis. Gr^low^ CD11b^hi^ immature myeloid cells in bone marrow and peritoneal cavity (**a** and **b**, respectivelly) and Gr^hi^ CD11b^hi^ mature granulocytes in bone marrow and peritoneal cavity (**c** and **d**, respectivelly) were quantified. In oil granulomas we observed an intense inflammatory infiltrate in both oil granulomas, however, in WT mice (**e**) this infiltrate was mainly composed by polymorphonuclear cells (insert), whereas in gal-3^−/−^ animals (**f**) it was formed by mononuclear cells (insert). Data are reported as means ± standard error and are representative of 5 animals of each group. (*, *P* < 0.05). Black bars: WT animals; white bars: gal-3^−/−^ animals

The increased cellularity in WT bone marrows was not accompanied by an increase in the number of myeloid progenitor cells (Gr-1^low^ CD11b^hi^ in Fig. 3a), but reflected an increase in the mature granulocytic population after 60 days (Gr-1^hi^ CD11b^hi^ in Fig. 3c). However, both of these populations were increased in peritoneal cavity, suggesting a recruitment of mature neutrophils to the initial site of the inflammation (Fig. 3b and d). In the absence of gal-3, we have observed a similar pattern of neutrophil mobilization (Fig. 3c and d), but significantly lower than in WT mice. An increase of granulocyte numbers in the peritoneal cavity and no cellularity modification in gal-3^−/−^ bone marrows suggest a bone marrow-independent cell recruitment to the site of inflammation.

**Fig. 4:**
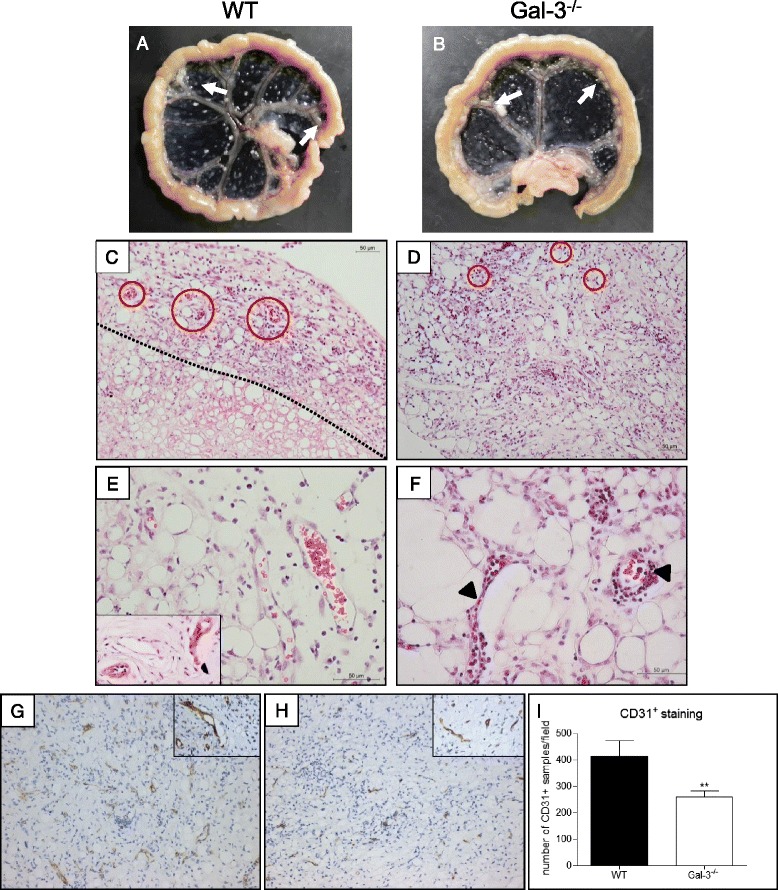
Oil granuloma development after pristane injection. Oil granulomas were formed in both groups of animals, though their organizations differed between them. After 60 days of pristane injection, oil granulomas can be macroscopically observed attached to the mesentery (white arrows), both in WT (**a**) and gal-3^−/−^ mice. **b** WT mice oil granulomas (**c**) were organized in cortical and medullary-like regions (*dotted line*) and were rich in blood vessels (**e** and *red circles* in **c**), while gal-3^−/−^ mice oil granulomas presented random distribution of cells (**d**) and few blood vessels (*red circles* in **d**), some of them surrounded by inflammatory cells (*arrows* in **f**). CD31^+^ staining for blood vessels confirmed the presence of these structures both in WT and gal-3^−/−^ mice (**g** and **h**, respectivelly). The staining intensity of CD31 was quantified in WT and gal-3^−/−^ mice (**i**). Data are representative of five animals of each group

As the bone marrow and peritoneal cavity compartments from both WT and gal-3^−/−^ mice showed different qualitative and quantitative cellular responses along pristane injection, we investigated the putative influence of these differences in the organization of oil granulomas. These structures can be macroscopically detected in the mesentery around 60 days after pristane injection, as we can observe in Fig. 4a and b.

As described above, the increased number of Gr^low^CD11b^hi^ myeloid progenitor cells and Gr^hi^CD11b^hi^ mature myeloid cells in the peritoneal cavity of WT mice (Fig. 3b and d, respectivelly) could reflect the cellular composition of oil granulomas in these mice. Indeed, Fig. 3e shows an intense presence of polymorphonuclear cells *foci* (arrows and insert), indicating a peripheral amplification inside the inflammatory site, in a time independent manner from the bone marrow production. In gal-3^−/−^ mice, oil granulomas were composed mainly by mononuclear cells, without the organization of polymorphonuclear *foci* (Fig. 3f, *arrowheads* and insert).

### Galectin-3 in granulomas induced by pristane differentially organizes cellular and extracellular components

Oil granulomas in WT animals, macroscopically examined at 60 days after pristane injection (Fig. 4a, *white arrows*), exhibited two distinct regions when histologically observed: a highly cellular cortical region (Fig. 4c, above *dotted line*) and an inner region less cellular, but rich in lipid material. The granulomas presented several blood vessels (Fig. 4c, *red circles*, and e), in many instances structured with a muscular tunica (Fig. 4e, insert). Macroscopic structures of gal-3^−/−^ mice oil granulomas were also obtained (Fig. 4b, *white arrows*). However, we did not observe a clear compartmentalization of the oil granulomas formed in gal-3^−/−^ mice. Instead, cells were randomly distributed, with blood vessels surrounded by inflammatory cells (Fig. 4d and f, *arrowheads*). Presence of blood vessels was confirmed by CD31 immunolocalization, as demonstrated in Fig. 4g and h. In WT mice, we observed a higher quantity of blood vessels, as observed in Fig. 4i, when compared to gal-3^−/−^ animals. These results suggest a favoring of cell mobilization process from both bone marrow and peritoneal cavity.

**Fig. 5:**
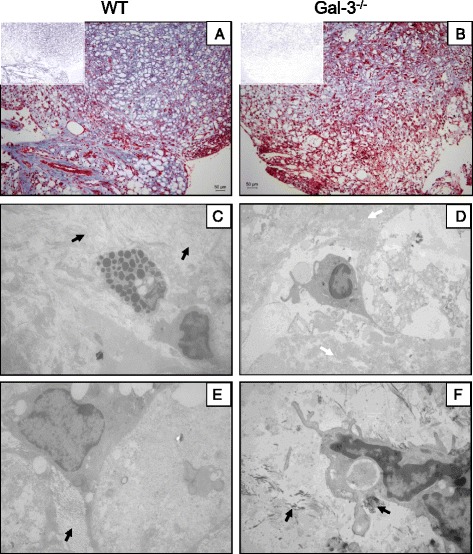
Oil granuloma extracellular matrix distribution and ultrastructure analysis. ECM could be observed in both WT and gal-3^−/−^ animals through trichrome staining (**a** and **b**, respectivelly), despite decreased in the absence of gal-3. Inserts represent the deconvolution of images to evidence the collagen fibers in blue. Transmission electron microscopy analysis revealed and confirmed these data. In WT mice a large amount of collagen fibers (**c** and **e**, *closed black arrows*) were observed in many directions. In gal-3^−/−^ animals oil granulomas presented lots of cell debris and dispersed ECM throughout the parenchyma (**d**, *closed white arrow*). Compared to WT granulomas, the structures formed in the absence of gal-3 presented great reduction of collagen fibers (**f**, *closed black arrows*)

It was also observed a difference in the distribution of extracellular matrix (ECM) components between WT and gal-3^−/−^ mice oil granulomas. In oil granulomas of WT mice, collagen was homogeneously distributed, presenting a more intense staining (Fig. 5a, insert) when compared to gal-3^−/−^ mice granulomas (Fig. 5b, insert).

We took advantage of the higher resolution of TEM to study the ultrastructure of granulomas. As previously observed, WT mice oil granulomas presented a large amount of randomly distributed collagen fibrils (Fig. 5c and e, *black arrows*). On the other hand, we observed by TEM the presence of large amounts of necrotic tissues and dispersed ECM in oil granulomas from gal-3^−/−^animals (Fig. 5d, *white arrows*). Furthermore, a drastic reduction in the amount of collagen fibrils was observed. They were dispersed in small quantities in the necrotic tissue, as demonstrated by trichrome staining (Fig. 5f, *black arrows*).

**Fig. 6:**
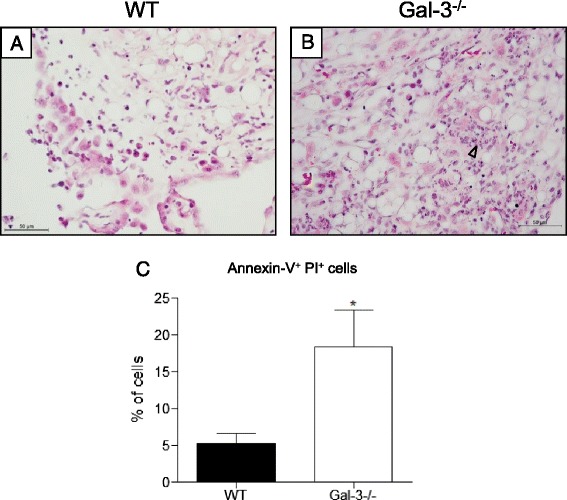
Cell death in oil granulomas structures. An intense presence of cell debris was observed in gal-3^−/−^ mice oil granulomas (**b**) compared to oil granulomas obtained from WT animals. **a** Phenotypic analysis of dissociated oil granulomas confirmed the increase of cell death observed in morphological data, with increase of annexin-V^+^ PI^+^ cells in gal-3^−/−^ compared to WT granulomas. **c** Data are reported as means ± standard error and are representative of five animals of each group. (*, *P* < 0.05). Black bars: WT animals; white bars: gal-3^−/−^ animals

In oil granulomas of gal-3^−/−^ animals we also observed an interesting phenotype: the presence of cell debris within the inflammatory parenchyma (Fig. 6b, *opened arrowheads*) when compared to WT mice (Fig. 6a). This result was corroborated after tissue dissociation, when we obtained a higher number of annexin-V^+^PI^+^ cells (Fig. 6c).

These data indicate that cellular and extracellular matrix composition inside inflammatory site of oil granulomas could negatively regulate bone marrow response, favoring a peripheral cellular amplification that may lead to different consequences to the outcome of the inflammatory process.

### The initial profile of cytokines produced by adherent peritoneal cells is the major responsible for the input of the inflammatory response

As we observed a drastic reduction of B lymphocytes in the absence of galectin-3, both in bone marrow and peritoneal cavity, and a differential oil granuloma organization between WT and gal-3^−/−^ mice, we investigated the expression of major pro-inflammatory cytokines by adherent peritoneal cavity cells. For this, we injected pristane in WT and gal-3^−/−^ mice and at 48 h or at 7 days we quantified cytokine mRNA levels expressed by the cells in the peritoneal cavity.

**Fig. 7:**
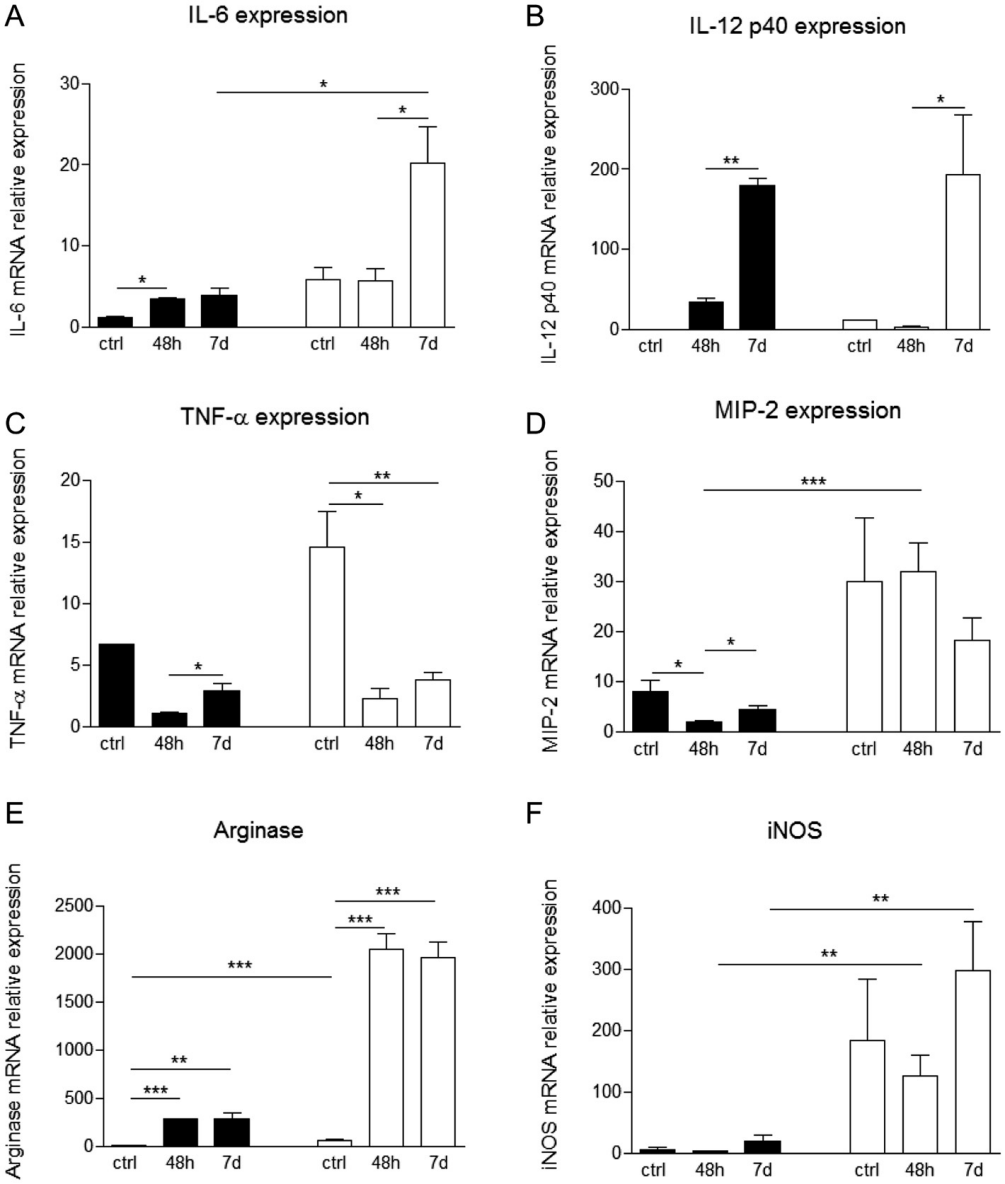
Expression of inflammatory cytokines by peritoneal adherent cells. Peritoneal adherent cells obtained from WT or gal-3^−/−^ animals injected for 48 h or 7 days with pristane or from non-injected mice were selected by plastic affinity and harvested for inflammatory cytokines gene expression analysis. IL-6 (**a**), IL-12 p40 (**b**), TNF-α (**c**), MIP-2 (**d**), arginase (**e**) and iNOS (**f**) were analyzed by RT-PCR. Data are reported as means ± standard error and are representative of five animals of each group. (*, *P* < 0.05). Black bars: WT animals; white bars: gal-3^−/−^ animals

The cytokine IL-6 is mainly produced by macrophages and is involved in the inflammatory processes and B cell differentiation. As we can observe in Fig. 7a adherent cells, obtained from gal-3^−/−^ mice at 7 days after pristane injection, presented an up regulation of mRNA levels for this cytokine when compared to WT mice. Adherent cells obtained from WT mice injected with pristane also presented an increase of IL-6 expression in comparison to control group, but proportionally lower (Fig. 7a). IL-12 p40 and TNF-α mRNA levels presented a different dynamics. While IL-12 p40 mRNA levels were up regulated during treatment in both WT and gal-3^−/−^ mice, TNF-α mRNA levels were gradually down regulated in the two groups of animals. However, both cytokines had expression levels upregulated in gal-3^−/−^ control mice when compared to WT control mice (Fig. 7b and c, respectivelly).

Macrophage inflammatory protein 2 (MIP-2), a cytokine related to neutrophil recruitment, was also analyzed. As we can observe in Fig. 7d, WT mice presented a down regulation of the mRNA levels at 48 h after pristane inoculation. In contrast, there was no significant difference in the mRNA levels in gal-3^−/−^. Curiously, when we compared the expression levels of mRNA of MIP-2 between WT and gal-3^−/−^ mice, this cytokine was more expressed in the absence of galectin-3 (Fig. 7d).

Expression of cytokines related to macrophage polarization, arginase and inducible nitric oxide synthase (iNOS), were analyzed in peritoneal adherent cells (Fig. 7e and f, respectivelly). Arginase expression presented the same pattern in WT and gal-3^−/−^ mice, increasing expression after 48 h of pristane stimulus, maintaining these levels increased until 7 days. However, this increase was significantly greater in the absence of galectin-3 when compared to WT animals (Fig. 7e). On the other hand, iNOS expression was not altered both in WT and gal-3^−/−^ mice during treatment. However, expression levels of iNOS in the absence of galectin-3 were increased in all time points analyzed when compared to WT animals (Fig. 7f).

## Discussion

In this study, our results indicate, for the first time, that differential granulomatous reaction and its components, including the presence of cellular debris, regulate mobilization and amplification of inflammatory cells from the bone marrow to the initial site of inflammation.

As observed in our data, oil granulomas formed in WT mice presented greater amounts of neutrophils distributed though parenchyma, followed by a very well organized structure, rich in blood vessels and a scaffold of connective tissue formed by collagen. As described in the literature [7, 28] these structures are formed by large amounts of lipid material, surrounded by inflammatory cells organized as lymphoid follicles, e.g., B lymphocytes nodules surrounded by T and dendritic cells. However, our data showed an intense infiltration of granulocytic cells in WT mice. These conflicting data may be due to the time frame after pristane stimulus chosen to be analyzed. Indeed, Nacionales’ group performed their analysis at least 12 weeks after pristane stimulus [7], whereas we analyzed after 8 weeks, using the same mice strain, eliminating possible genetic background influence. However, Chen et al. demonstrated that genetic background contributes to organization and composition of oil granulomas, raising conflicting data in the literature [14, 28]. Thus, both time frame and genetic background can influence separately or synergistically the development of oil granulomas.

On the other hand, oil granulomas formed in gal-3^−/−^ animals were mainly composed by mononuclear cells, including B and T lymphocytes, and reduced amounts of neutrophils. These structures presented low vascularization and ECM, which likely promoted intense cell death. The reduced amounts of neutrophils in peritoneal cavity of gal-3^−/−^ mice could reflect the delay in myeloid cell maturation [27] or a delayed migration from the bone marrow [24], as described in the literature. Schauer and Gresnigt groups have described an important role of neutrophils in resolution of the inflammatory process by reducing proinflammatory cytokines production [29, 30]. In view of these results, it is important to analyze in our model the role of neutrophils in late stages of pristane-induced inflammation and their outcome to plasmacytoma and/or autoimmune diseases development.

The observation of an increase in the expression levels of MIP-2 in gal-3^−/−^ mice, an important cytokine involved in neutrophils recruitment [31, 32], suggests that peritoneal macrophages upregulate MIP-2 in an attempt to compensate the reduced recruitment of neutrophils to inflammatory site or the excess of cell death observed in these animals.

Galectin-3 plays an important role as a negative regulator in apoptotic events [26, 33], so that oil granulomas deficient in this lectin presented a great number of cells positively stained for annexin-V, apoptotic cells and great amounts of cell debris. The persistence of apoptotic cells and cell debris *in situ* could also be a result of low phagocytic state of macrophages, as our group previously described in gal-3^−/−^ mice [26].

The increase in the expression levels of IL-12 p40 and IL-6 expression is already described in the literature as negative regulator of B1 B lymphocytes [34]. As we observed in our data, in the absence of galectin-3, IL-6 expression in adherent cells was significantly upregulated 7 days after pristane injection. In addition, there was a drastic reduction of B1 lymphocytes in the peritoneal cavity. Interestingly, these same parameters were observed in WT mice only 60 at days after pristane injection. Both IL-6 and IL-12p40 upregulation were observed in WT and gal-3^−/−^ mice. On the other hand, TNF-α down modulation was also observed in WT mice, corroborating data described by Chae and Shin [35], however, this reduction was more drastic in the absence of galectin-3. Adherent cells of WT mice expressed high levels of arginase and presented no differences in iNOS expression after pristane injection, suggesting polarization of macrophages to an anti-inflammatory M2 phenotype. In the gal-3^−/−^ mice, we also observed polarization to M2 phenotype coupled to unchanged levels of no iNOS expression. However, since these animals have high expression of iNOS in the absence of inflammatory stimulus, we suggest that this initial pro-inflammatory M1 background could disturb the response, maintaining the inflammatory status and promoting a rise in cell death within the oil granulomas.

Bone marrow compartment is a productive site of hematopoietic cells, which are continuously mobilized to the primary site of inflammation, and contribute to the development and organization of oil granulomas. In our model, as expected, bone marrow cellularity clearly increased in WT mice after 60 days of pristane injection. An increased cellularity was also observed in the peritoneal cavity fluid, providing evidence that the bone marrow cells are migrating to the initial site of inflammation. However, galectin-3 deficient mice seem not to respond in the same way to the pristane injection, since the cellularity in both bone marrow and peritoneal cavity of these animals was not altered during the experiments. Despite the increase in the total number of mature neutrophils, both in bone marrow and peritoneal cavity, at 60 days after pristane injection, the total number of granulocytic precursors (Gr^low^CD11b^hi^) remained unchanged in bone marrow. On the other hand, these precursors were significantly increased in the peritoneal cavity, suggesting a local amplification of these cells. It likely occurred in oil granulomas and in large scale in WT mice, since we observed greater quantities of mature neutrophils in these animals, and more myeloid clusters inside the oil granulomas.

As related in the literature, pristane can be toxic to cells inducing apoptosis and necrosis [17, 36]. In addition, it can accumulate in hematopoietic organs, like spleen and bone marrow [37]. Our group has previously demonstrated that in the absence of galectin-3, lymph node cells are more susceptible to apoptosis [26]. Bone marrow cells of galectin-3 deficient mice might be more sensitive to pristane toxicity, explaining why cellularity in this organ did not increase.

## Conclusions

Our data indicated that the first steps of oil granuloma development, with a local amplification of inflammatory myeloid cells, led to a gradual reduction of bone marrow cells mobilization, turning the local responses more autonomous. In contrast, the altered *milieu* of oil granulomas (low amount of ECM and high of cell debris) could disturb the bone marrow physiology in an attempt to compensate the absence of peripheral amplification. This altered microenvironment of gal-3^−/−^ oil granulomas could be partially explained by an unbalance of pro and anti-inflammatory cytokines produced by macrophages.

Our next steps are to evaluate the consequences of normal and necrotic oil granulomas in the development of lymphoproliferative diseases such as plasmacytoma, which is classically associated with long lasting chronic inflammatory processes.

## Methods

### Animals

Galectin-3 deficient mice (gal-3^−/−^) were generated as described [24] and backcrossed to BALB/cJ mice for nine generations. Age and sex matched wild type (WT) mice on BALB/cJ background were used as controls and were obtained from a colony bred at Federal University of São Paulo (Brazil). All mice procedures were performed at Federal University of Rio de Janeiro in accordance with institutional guidelines (protocol number DAHEICB071, Federal University of Rio de Janeiro). To induce inflammatory process, 8–12 weeks-old mice received a single intraperitoneal injection of 500 μL of 2,6,10,14 tetrametyl-pentadecane (pristane) (95 % pure, Sigma-Aldrich, St Louis, MO, USA). After 7 or 60 days, mice were euthanized using a carbon dioxide chamber. Non injected mice were used as controls.

### Histological preparations

For histological analysis, WT and galectin-3^−/−^ mice were sacrificed 60 days after pristane injection. Several oil granulomas were excised, washed in cold saline, fixed in buffered 10 % formalin for 24 h at 4 °C, embedded in paraffin and sectioned into 4-m thick slices. The slices were dewaxed using xylene, hydrated in a graded ethanol, and then washed with distilled water. Lastly, the slices were processed routinely with hematoxylin & eosin (H&E) or with Masson’s Trichrome stain. For Masson’s Trichrome stain, briefly, slides were placed in Bouin solution for 1 h at 56 °C, then stained with Weigert hematoxylin (10 min; Sigma-Aldrich, St. Louis, MO), followed by Biebrich scarlet acid fuchsin (5 min; Sigma-Aldrich), phosphomolybdic/phosphotungstic acid solution (10 min; Sigma-Aldrich), and aniline blue (5 min; Sigma-Aldrich).

### Transmission electron microscopy (TEM)

Oil granulomas were fixed in a solution containing 2.5 % glutaraldehyde, 4 % formaldehyde and 0.1 M sodium phosphate buffer for 4 h at room temperature. Part of the granulomas was cut in two halves to allow the fixative to diffuse faster throughout the granulomas. After rinsing in the same buffer (3 × 10 min), the samples were dehydrated in graded series of acetone till absolute and embedded in epoxy resin (Embed 812). Ultrathin-sections (70 nm) were obtained using a ultramicrotome (Leica) and a diamond knife (Diatome), collected in 300 mesh copper grids and stained with 5 % uranyl acetate for 30 min and 1 % lead citrate for 3 min. The sections were observed in a Zeiss 900 TEM operated at 80 kV and images (2048 × 2048 pixels) acquired using a CCD camera (Olympus).

### Immunohistochemistry

Paraffin-embedded sections were de-waxed and hydrated. After inhibition of endogenous peroxidase, sections were incubated for 1 h with 0.01 M PBS containing 5 % BSA, 4 % skim milk, 0.1 % Triton X-100 (Sigma Aldrich, USA), 0.05 % Tween-20, and 10 % normal goat serum followed by incubation with purified antibodies overnight at 4 °C in a humid chamber. Primary antibodies were revealed with a biotinylated anti-rat IgG (BA-4001, Vector Laboratories, Burlingame, CA, USA) and developed with avidin-peroxidase (1:200 in PBS) (Sigma Aldrich, USA), using diaminobenzidyne as the chromogen. Sections were counterstained with Harris’ hematoxylin.

### Cell suspensions

Bone marrow cells were obtained *ex vivo* by flushing the femoral cavity of WT and gal-3^−/−^ mice with phosphate buffered saline (PBS), pH 7.2, with 3 % fetal bovine serum (FBS, LGC; Cotia, São Paulo, Brazil). Peritoneal cells were harvested on cold PBS with 3 % FBS. A pool of oil granulomas was obtained from each animal, and cells were recovered by digesting the tissues with 2 mg/mL type I-A collagenase and 1 mg/mL dispase in DMEM (Sigma-Aldrich) with constant stirring for 1 h at 37 °C. The resulting cell suspension was removed and filtered through fine nylon mesh [7]. Cells were washed, counted and prepared for further experimentation. When indicated, red blood cell lyses were done with hypotonic ACK (ammonium chloride potassium) solution.

### Flow cytometry

In order to saturate Fc receptors, 1 × 10^6^ cells/mL bone marrow, peritoneal and oil granuloma-derived cells were incubated with the Fc blocker antibody, produced by clone 2.4G2 (obtained from the Rio de Janeiro Cell Bank PABCAM, Federal University, Rio de Janeiro, Brazil), for 10 min before adding specific monoclonal antibodies (mAbs). Cells were incubated with mAbs for 30 min; unbound mAbs were washed out with PBS. The following mAbs were used: for T lymphocytes quantification, triple staining was used (FITC-labeled CD8, PE-labeled CD4 and PerCP-labeled CD5); for peritoneal cavity B lymphocytes quantification, double staining was used (FitC-labeled CB11b and APC-labeled B220); for bone marrow B lymphocytes analysis a single staining was used (APC-labeled B220), since there was not observed any double positive (B220+ CD11b+) B1 cells; and for mature and precursor granulocytes analysis a triple staining was used (FitC-labeled CD11b, PerCP-labeled Gr-1 and APC-labeled Ly-6G), where double high positivity for Gr-1 and CD11b was characterized as mature granulocytes, as emphasized by Ly-6G staining, and Gr-1 low and CD11b high cells were characterized as myeloid progenitors. All gating strategies used in this paper are demonstrated in Additional file 1: Figure S1. All antibodies used were obtained from BD Bioscience, San Jose, CA, USA. For cell death analysis, cells were incubated with Annexin-V FITC, according to manufacturer informations (BD Bioscience). Samples were acquired in a flow cytometer (FACScalibur; BD Bioscience) using Cell Quest software and analyzed using WinMDI 2.9 software.

### Isolation of adherent peritoneal cells

Animals were divided into four groups with five animals each: (1) WT control group: this group was injected with 500 μL of PBS; (2) WT pristane-treated group: received a single intraperitoneal injection of 500 μL of pristane; (3) gal-3^−/−^ control group: this group was injected with 500 μL of PBS; (4) gal-3^−/−^pristane-treated group: received a single intraperitoneal injection of 500 μL of pristine. At the end of the experimental period (48 h and 7 days), animals were sacrificed and peritoneal lavage samples were collected by injecting 3 mL of phosphate-buffered saline (PBS). Individual lavage samples were cultured in a 6 well plate at 37 °C with 5 % CO_2_. After 2 h, peritoneal adherent cells were rinsed with PBS to remove non-adherent cells and subsequently, total RNA was isolated using Tri-Reagent (Sigma).

### Total RNA extraction, reverse transcription and quantitative PCR

Total RNA from peritoneal adherent cells cultures was isolated using the Tri-Reagent (Sigma) according to the manufacturer’s instructions. Complementary DNA (cDNA) was synthesized from 1 μg of total RNA using the High capacity cDNA RT kit (Applied Biosystems), according to the manufacturer’s protocols. Quantitative PCR analysis was performed in triplicate using Power SYBR Green Master Mix (Applied Biosystems). Relative quantification was done using the ΔΔCt method normalizing to β-actin gene expression. For primer sequences see following Table 1.

**Table 1:** Primer sequences

Gene	Primer sequence (5’-3’)
β-actina	Forward: AGCTGCGTTTTACACCCTTT
	Reverse: AAGCCATGCCAATGTTGTCT
IL-12p40	Forward: AACCATCTCCTGGTTTGCCA
	Reverse: CGGGAGTCCAGTCCACCTC
iNOS-2	Forward: CCGAAGCAAACATCACATTCA
	Reverse: GGTCTAAAGGCTCCGGGCT
Arginase-1	Forward: CAAAAGGACAGCCTCGAGGAG
	Reverse: CCCGTGGTCTCTCACGTCAT
IL-6	Forward: TCAATTCCAGAAACCGCTATGA
	Reverse: GAAGTAGGGAAGGCCGTGGT
TNF-α	Forward: GACGTGGAACTGGCAGAAGAG
	Reverse: GCCACAAGCAGGAATGAGAAG
MIP-2	Forward: ATCCAGAGCTTGAGTGTGACGC
	Reverse: AAGGCAAACTTTTTGACCGCC
Cxcl-1	Forward: AAAAGGTGTCCCCAAGTA
	Reverse: AAGCAGAACTGAACTACCATCG

### Statistical analysis

The statistical tests were applied using Tukey’s multiple comparison test (*t* test) and the significance threshold was fixed for α = 0.05. Therefore, P values ≤0.05 were considered statistically significant.

## Additional files

Additional file 1: Figure S1. Gating strategies to analyze lymphoid and myeloid subpopulations – To analyze T lymphocytes a triple staining strategy was used with CD5, CD4 and CD8 antibodies. First, all CD5^+^ cells were gated, and then, CD4 and CD8 expression was analyzed in this R1 population, characterizing CD5^+^CD4^+^ and CD5^+^CD8^+^ T lymphocytes (A). To quantify peritoneal B cells, a double staining strategy was used with B220 and CD11b antibodies, where B220^+^CD11b^−^ cells were considered B2 lymphocytes and B220^+^CD11b^+^ cells were considered B1 lymphocytes (B). And, finally, to characterize myeloid cells, a triple staining strategy was used with CD11b, Gr-1 and Ly-6G antibodies. All CD11b^high^ Gr-1^high^ cells are neutrophils, as observed by Ly-6G expression, and cells which expressed low levels of Gr-1 (CD11b^high^ Gr-1^low^ cells) were considered myeloid progenitors (C). (PDF 53 kb)
