# Supplementary figures and images for: Differential development of oil granulomas induced by pristane injection in galectin-3 deficient mice
Source: BMC Immunol. 2015 Nov 14;16:68. doi: 10.1186/s12865-015-0133-9 (PMC4647586; doi:10.1186/s12865-015-0133-9)

# SUPPLEMENTARY FIGURE 1

A

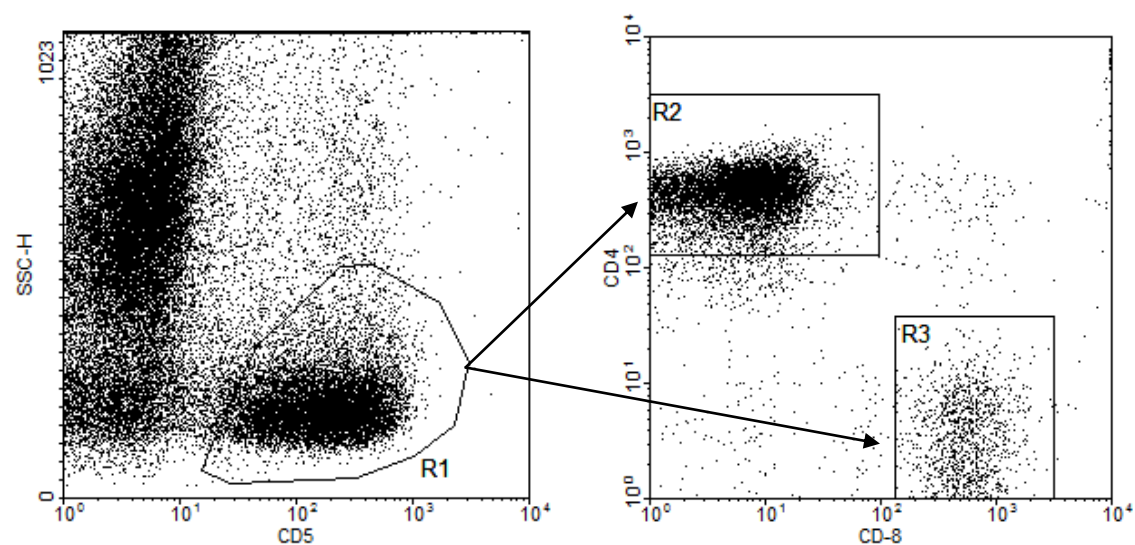

B

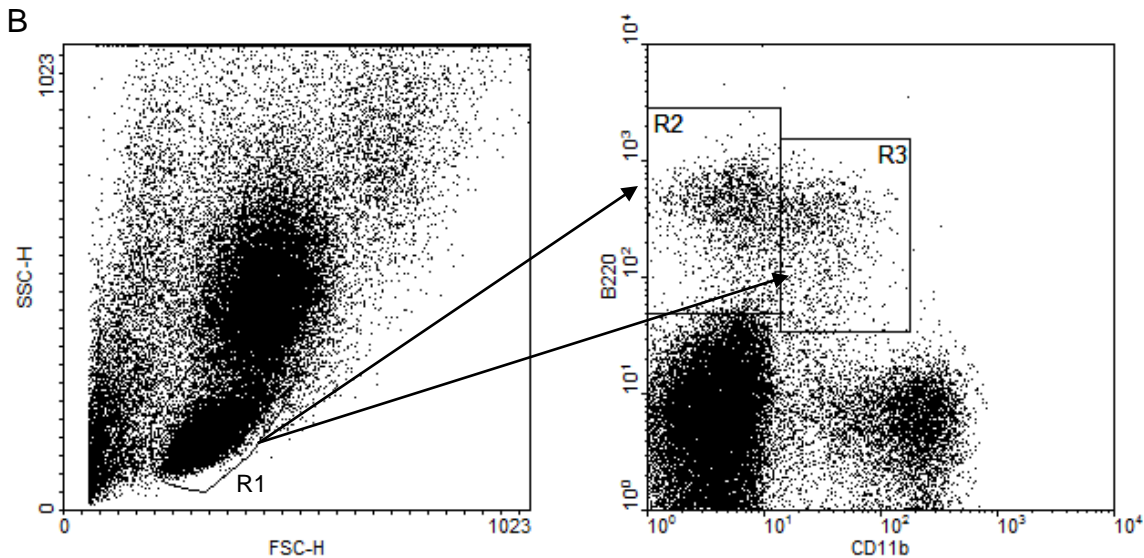

C

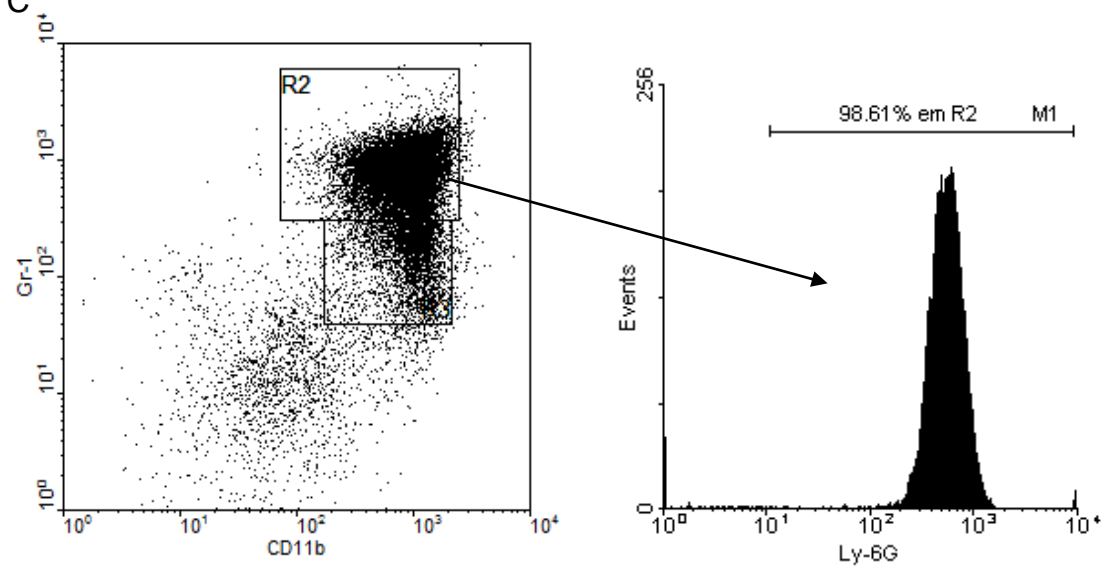

Supplement: Additional file 1: Figure S1. — Gating strategies to analyze lymphoid and myeloid subpopulations – To analyze T lymphocytes a triple staining strategy was used with CD5, CD4 and CD8 antibodies. First, all CD5+ cells were gated, and then, CD4 and CD8 expression was analyzed in this R1 population, characterizing CD5+CD4+ and CD5+CD8+ T lymphocytes (A). To quantify peritoneal B cells, a double staining strategy was used with B220 and CD11b antibodies, where B220+CD11b− cells were considered B2 lymphocytes and B220+CD11b+ cells were considered B1 lymphocytes (B). And, finally, to characterize myeloid cells, a triple staining strategy was used with CD11b, Gr-1 and Ly-6G antibodies. All CD11bhigh Gr-1high cells are neutrophils, as observed by Ly-6G expression, and cells which expressed low levels of Gr-1 (CD11bhigh Gr-1low cells) were considered myeloid progenitors (C). (PDF 53 kb) [file 12865_2015_133_MOESM1_ESM.pdf]
